# Influence of Fe_2_O_3_, MgO and Molarity of NaOH Solution on the Mechanical Properties of Fly Ash-Based Geopolymers

**DOI:** 10.3390/ma15196965

**Published:** 2022-10-07

**Authors:** Brăduț Alexandru Ionescu, Mihail Chira, Horațiu Vermeșan, Andreea Hegyi, Adrian-Victor Lăzărescu, Gyorgy Thalmaier, Bogdan Viorel Neamțu, Timea Gabor, Ioana Monica Sur

**Affiliations:** 1 NIRD URBAN-INCERC Cluj-Napoca Branch, 117 Calea Floresti, 400524 Cluj-Napoca, Romania; 2 IOSUD UTCN Doctoral School, Technical University of Cluj-Napoca, 15 Daicoviciu Street, 400020 Cluj-Napoca, Romania; 3 Faculty of Materials and Environmental Engineering, Technical University of Cluj-Napoca, 103-105 Muncii Boulevard, 400641 Cluj-Napoca, Romania

**Keywords:** geopolymer, compressive strength, Fe_2_O_3_, MgO

## Abstract

The use of waste from industrial activities is of particular importance for environmental protection. Fly ash has a high potential in the production of construction materials. In the present study, the use of fly ash in the production of geopolymer paste and the effect of Fe_2_O_3_, MgO and molarity of NaOH solution on the mechanical strength of geopolymer paste are presented. Samples resulting from the heat treatment of the geopolymer paste were subjected to mechanical tests and SEM, EDS and XRD analyses. Samples were obtained using 6 molar and 8 molar NaOH solution with and without the addition of Fe_2_O_3_ and MgO. Samples obtained using a 6 molar NaOH solution where Fe_2_O_3_ and MgO were added had higher mechanical strengths compared to the other samples.

## 1. Introduction

One of mankind’s most important problems is climate change, which is occurring as a result of the significant increase in annual temperatures [1]. Anthropogenic industrial activities release large amounts of greenhouse gases such as carbon dioxide (CO_2_) into the atmosphere, which adversely affect the climate [2]. The construction industry is one of the main sources of greenhouse gas emissions, accounting for half of global emissions [3].

After water, concrete is the most widely used material in the world, with annual production exceeding 20 billion tonnes [4,5,6,7]. Portland cement is the most widely used powder to bind different constituents of concrete. According to a report, about 4.2 billion tonnes of Portland cement were manufactured in 2016 just to meet the high market demand [8]. Energy consumption for Portland cement manufacturing is estimated at 3% of global energy consumption [9]. In addition, one tonne of Portland cement production causes one tonne of CO_2_ emissions, and the cement industry contributes with 7–8% of global CO_2_ emissions [10,11]. Due to high emissions and energy consumption, the Portland cement industry is considered one of the main causes of climate change and contributes about 65% to global warming [12]. Approximately 1.5 tonnes of virgin raw materials are used to manufacture one tonne of Portland cement, leading to the depletion of natural resources [13,14]. Climate experts suggest that mankind should reduce emissions to zero by 2050 to limit global warming to 1.5 °C [1]. Therefore, scientists are focusing on limiting Portland cement production by finding environmentally friendly and energy efficient concrete binders. A first step is to substitute a quantity of Portland cement with fly ash and obtain concrete with self-healing properties [15,16].

One of the most important alternative binders to Portland cement are geopolymers also called inorganic polymers, consisting of alternating tetrahedral chains of SiO_4_ and AlO_4_, connected by a common oxygen atom and balanced by cations [17,18]. Some precursor materials used to produce geopolymers contain large amounts of iron. Although the presence of iron could play an important role in the structure and properties of geopolymers, Al substitutions with Fe have not yet been fully studied, even though they might occur in clays [19,20,21]. For example, fly ash, with an iron content of about 10%, stands out among these commonly used iron-rich precursor materials and up to 40% for some low-calcium ferric slag materials [22]. The compressive strength of samples obtained with these types of materials ranged from 20 to 80 MPa.

Studies on geopolymers are largely based on traditional precursor materials such as metakaolinite (2% Fe_2_O_3_ content), fly ash (10% Fe_2_O_3_ content) and blast furnace slag (0.5% Fe_2_O_3_ content). However, recent studies have shown that precursors with higher iron content than typically found in fly ash can be activated in alkaline mediums [20,21] with engineering applications. The presence of iron trioxide in the heat-treated geopolymer paste results in the formation of a ferro-silicate geopolymer [-Fe-O-Si-O-Al-O]. The amount of substituted Fe atoms can vary between 5% and 50% of the total amount of Fe_2_O_3_ contained in the geopolymer binder [23].

Additionally, several studies [23,24,25,26,27] indicate that binders and concrete obtained from alkali-activated slag show high mechanical strength and good performance to chemical attack, freeze–thaw cycles and high temperatures.

However, previous research [28,29,30,31] has shown that alkali-activated slag mortar and concrete is subject to substantial shrinkage by drying. This is one of the main disadvantages of the definitive use of alkali-activated slag as an alternative to traditional Portland cement binders. There are a number of factors that determine the drying shrinkage of alkali-activated slag, including the type and content of alkali activators [30,32,33,34], aggregate and slag properties [28,35], and the curing environment [36,37,38,39].

In general, sodium silicate-activated slag has higher shrinkage than sodium hydroxide-activated slag, and the drying shrinkage of alkali-activated slag increases with increasing activator dosage as well as with slag fineness [34,40]. In addition, the shrinkage of alkali-activated slag is very sensitive to the curing medium.

The use of magnesium oxide, MgO, as a shrinkage-reducing mineral additive dates back to the mid-1970s. Volume compensation during the drying process was due to the chemical reaction between MgO and water forming brucite (Mg(OH)_2_), which results in a 118% increase in volume [41]. The effect of MgO in alkaline-activated slag systems has been investigated recently, either in terms of its naturally variable content in different slag compositions [42] or as an additive [43]. Ben Haha et al. [42] investigated the effect of the natural MgO content in different slags on the performance of alkali-activated slag and showed that although the main hydration product is still C-S-H gel, MgO reacts with slag to form hydrotalcite (Mg_6_A_l2_(OH)_16_CO_3_•4H_2_O), the content of which increases as the MgO content of the slag increases. They also concluded that because these hydrotalcite-like phases are bulkier than C-S-H, they lead to higher strength, therefore the higher the MgO content, the higher the strength. In the work of Fei Jin et al. [44], the effect of adding MgO as a commercial reagent on the drying shrinkage and strength of alkali-activated slag was studied. It was found that MgO with high reactivity accelerated the early hydration of alkali-activated slag, while MgO with medium reactivity had little effect. Drying shrinkage was significantly reduced by highly reactive MgO, but cracking resulted after drying the samples. On the other hand, MgO with medium reactivity caused a reduction in shrinkage only after one month, but cement strength was improved.

In general, as the concentration of NaOH solution increases, the compressive strengths of samples obtained by alkali-activation of fly ash increase, but there are situations where the strength decreases. This variation in the effect of NaOH concentration on compressive strength is probably due to the different nature and type of molecules that form the fly ash particles. These differences between the types of molecules affect the degree of leaching of SiO_2_, Al_2_O_3_, CaO and Fe_2_O_3_ in the alkaline activator, where SiO_2_ leaching is slower than the other components [45]. According to Fernández-Jiménez and Palomo [46], NaOH concentration is responsible for the decomposition of the bonds of the main oxides. Thus, fly ash with higher SiO_2_ content requires a higher NaOH concentration to release SiO_2_ and the other oxides from fly ash particles to initiate geopolymerization.

The presence of coarser particles in the fly ash reduces the surface area that is exposed to the alkaline activator [47]. This means that a low chemical reaction and partial dissolution may occur on the surface of the coarse particles. As a result, these unreacted particles will be a weak point in the geopolymer matrix, which consequently reduces the compressive strength of the geopolymer specimens.

In terms of the mechanism of geopolymerization reactions, it is currently estimated worldwide that this process is a result of the dissolution of Si_2_O_3_ and Al_2_O_3_ oxides into atoms under the influence of the Na^+^ and hydroxyl (OH^−^) ion supplying alkali activator. These dissolved atomic species of Si and Al, in the presence of water, form a gel in which the atoms move freely, allowing the formation of monomers, followed by poly- and oligomerization, finally leading to the formation of three-dimensional chain networks. In contrast to the hydration-hydrolysis mechanism specific to Portland cement, geo-polymerization expels water in the polymerization/hardening/maturation process. This process is called “dehydroxylation”, water having only the function of facilitating the mobilities of the constituent groups in the gel matrix to form the specific bonds, the whole process can be represented in a generalized equation of the form (Equation (1)) [48,49]:

(1) $$\left[R\right]-O-\mathrm{Si}-\mathrm{Si}-\left(\mathrm{OH}\right)\;\left[R\right]-\mathrm{Si}-O-\left[r\right]+H_{2}O$$

where:

[R] = atoms connected to -O-Si-OH, (Al or Fe)

[r] = new chain sequences that connect to [R]-Si-O- to form a larger chain

“+H_2_O” indicates the expulsion of water for the bonds to form. The geopolymer acquires its strength by creating long networks of three-dimensional chains, leading to the initial use of a large amount of capillary H_2_O, followed by its expulsion once a suitable bond can form.

Worldwide, in the general study on the production of geopolymer binders, there are still a number of controversies or insufficiently clarified elements, as their mechanical strengths and other physical-mechanical performances are strongly influenced by the oxide composition of the main raw materials and additives, the type and molarity of the alkali activators, the existence or not of heat treatment. From the point of view of the oxide composition and other characteristics of the raw materials, depending on their origin, there is a great heterogeneity, which is the main difficulty in the production of geopolymer binders, the customization of the mixtures by the mass ratio of raw materials and the molarity of the alkaline activator, from case to case, being essential.

The aim of this paper is to analyse the possibility of producing geopolymer binders and the influence of Fe_2_O_3_ and MgO additions and the molarity of the NaOH solution on its mechanical strengths, under the conditions of using a local fly ash, specific for Romanian thermal power plants.

## 2. Materials and Methods

### 2.1. Materials

The raw materials used in this study for producing alkali-activated geopolymer pastes were selected locally and consisted of fly ash (F.A.), iron trioxide (Fe_2_O_3_), magnesium oxide (MgO), sodium hydroxide solution (6M, respectively, 8M) and sodium silicate solution Na_2_SiO_3_ 34%.

Fly ash used in the production of the geopolymer binder was obtained from the Rovinari Thermal Power Plant, Romania. Iron trioxide, magnesium oxide, sodium silicate and NaOH in the form of micropearls with 99.7% purity were purchased commercially. The chemical composition of the fly ash is shown in Table 1.

### 2.2. Synthesis of Geopolymer

**Figure 1:**
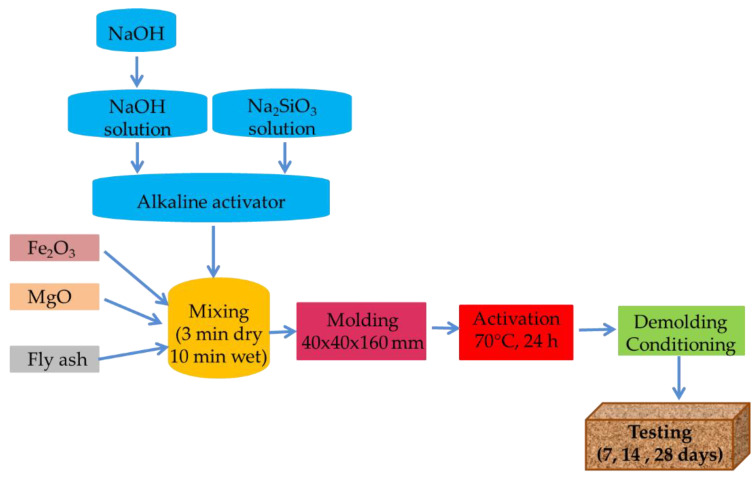
Methodology used for the preparation of the geopolymer paste.

Two geopolymer pastes were prepared from fly ash, and an alkaline activating solution by combining NaOH solution (6M and 8M, respectively) with Na_2_SiO_3_ solution and two geopolymer pastes to which iron trioxide and magnesium oxide were added in addition to ash. The ratio of sodium silicate solution to Na_2_SiO_3_/NaOH sodium hydroxide solution was set to 2. The procedure used in the production of the alkali-activated fly ash based geopolymer binders with added iron trioxide and magnesium oxide is shown in Figure 1. The mixing of fly ash with iron trioxide and magnesium oxide was done for 3 min for complete homogenization using a paddle mixer. Subsequently, after mixing the fly ash with the iron trioxide and magnesium oxide, the alkali activator solution was poured in gradually over 70 s, initially at low speed. Pouring the alkaline activator too abruptly can lead to instant curing effect. The mixing of alkaline activators with fly ash, iron trioxide and magnesium oxide was done for 10 min. After mixing the obtained mixture was poured into rectangular moulds with inner dimensions of 40 × 40 × 160 mm. The geopolymer mixture was kept in the oven for 24h at an activation temperature of 70 °C. After removal from the oven the obtained samples were kept in the climate chamber at a temperature of 23 °C and relative humidity of 50% and their flexural and compressive strengths were measured at 7, 14 and 28 days.

Four geopolymer paste mixtures were prepared and investigated, two with NaOH solution (6M) and two with NaOH solution (8M). The mass ratio of sodium silicate solution to sodium hydroxide solution was set to 2, and the solution mass to dry mass ratio was 0.9. The proportions of substances used for the preparation of the four recipes are shown in Table 2. In order to understand the influence of Fe_2_O_3_, MgO and the molarity of the NaOH solution, 1% Fe_2_O_3_ and 1% MgO was added to the amount of ash used. This addition of Fe_2_O_3_ and MgO was done for both a NaOH solution molarity of 6M and a molarity of 8M.

The literature indicates the possibility of using NaOH solution for the preparation of alkaline activator, with various molarities, to obtain geopolymeric materials. For economic and environmental impact reasons, but also to create a basis for preliminary analysis for further research, using NaOH solutions with higher molarity, in this study the 6M and 8M variants were chosen.

Preliminary investigations carried out only with the addition of 1%, 5% and 10% iron trioxide (Fe_2_O_3_) in relation with mass ratio of fly ash and 6M NaOH solution molar concentration used for the production of the alkaline activator, showed that, in terms of mechanical strengths, as the amount of Fe_2_O_3_ increased, they decreased by up to 3.3%. Therefore, for further research, 1%, in relation to the amount of ash, was considered as the optimum addition of Fe_2_O_3_.

Similarly, for the sodium hydroxide solution with 6M molar concentration, the compressive strength of the samples produced using 1% Fe_2_O_3_ + 1% MgO, 1% Fe_2_O_3_ + 5% MgO and 1% Fe_2_O_3_ + 10% MgO were analysed. The experimental results showed a reduction of this parameter by up to 16% (compressive strength of the mixtures with 1% Fe_2_O_3_ + 10% MgO compared to that of the mixtures with 1% Fe_2_O_3_ + 1% MgO). Therefore, for further research, 1%, in relation to the amount of ash, was considered as the optimal addition of Fe_2_O_3_ and 1%, in relation to the amount of ash, as the optimal addition of MgO.

Based on preliminary results obtained for the situation using 6M NaOH solution, the hypothesis of preservation of the trend of evolution of the compressive strength in the variant using 8M NaOH solution was verified. The negative influence of excess addition of oxides was also observed, so the mixtures presented in Table 2 were established in order to analyse the influence of the molarity of the NaOH solution on the geopolymer mechanical performances.

Crystal structure analysis of the layers was performed by X-ray diffraction (XRD) using a high-resolution Brucker D8 diffractometer with copper anode (CuKα1 = 1.54056 Ǻ). X-ray diffraction (XRD) was used to estimate structural and microstructural properties.

Morphological and microstructural characterization was performed using scanning electron microscopy (SEM) and energy dispersive spectroscopy (EDS)). A JEOL JSM 5600 LV (JEOL, Ltd., Tokyo, Japan) high-resolution scanning electron microscope (SEM) equipped also with an electron back-scattered diffraction detector (EBSD) was used in the present work.

The evolution of a three-dimensional shrinkage phenomenon was observed during the 7 days of specimen conditioning, prior to testing in terms of mechanical characteristics, and the shrinkage was evaluated as a volume reduction in relation to the initial volume (40 × 40 × 160 mm). Uniaxial bending and compressive strength were measured using the Advantest 9 testing machine (Advantest Corporation, Tokyo, Japan).

Flexural strength was determined using the three-point bending test (3PB) in accordance with EN 196-1. A digital flexural strength tester suitable for loads up to 10 kN (±10%) with a loading rate of (50 ± 10) N/s was used. The testing machine is provided with a bending device consisting of two steel support rollers with a diameter of (10 ± 0.5) mm, arranged at a distance of (100 ± 0.5) mm from each other and a third load roller, placed centrally.

The compressive strength of the specimens was determined in accordance with EN 196-1, using the compression test of the prismatic specimen halves resulting from the three-point bend test (3PB). The compressive loading rate used was 50 N/s (0.12 MPa/s). Samples were tested at 7, 14 and 28 days of age.

Experimental testing was carried out under laboratory conditions, ensuring compliance with repeatability and reproducibility requirements.

## 3. Results and Discussions

The experimental results show both the influence of the addition of Fe_2_O_3_ and MgO oxides and the molarity of the NaOH solution used for the preparation of the alkaline activator on the physical-mechanical performance of the geopolymer material. From the beginning, a volume shrinkage of the tested specimens between 1.5% and 5.0% was recorded. This can be attributed to the water content in the mixture, which is directly influenced by the molarity of the NaOH solution, water that is removed in the heat treatment process.

### 3.1. Flexural Strength

**Figure 2:**
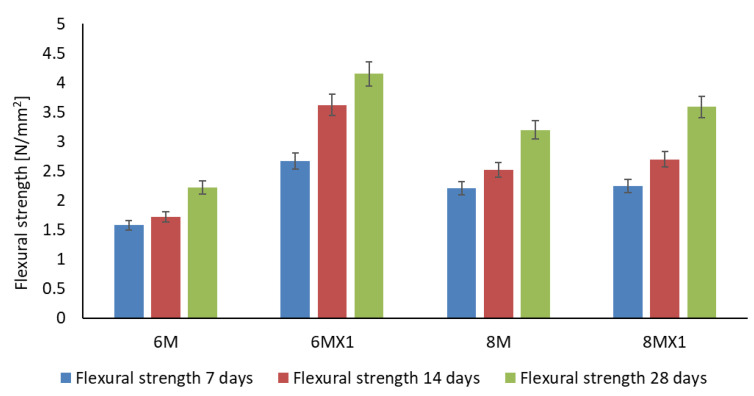
Flexural strength of the alkali-activated geopolymer samples.

Analysing Figure 2 shows an increase in flexural strength of all samples from 7 days to 14 days of age. It is also found that the samples containing Fe_2_O_3_ and added MgO have a higher strength than the control samples. For example, for a 6M molarity of NaOH solution, the sample (6MX1) containing 1% Fe_2_O_3_ and 1% MgO added to the ash used, has a flexural strength of more than 69% compared to the sample (6M) containing no additional elements, at 7 days, over 110% at 14 days and over 80% at 28 days. Similarly, but less obvious, for a molarity of 8M NaOH solution, we have an increase in the bending strength of the 8MX1 sample of 1%, 7% and 12% at 7, 14 and 28 days, respectively. The flexural strength of sample 6MX1 has the highest value compared to the other samples. Although the molarity of the NaOH solution for sample 8MX1 is higher than that of sample 6MX1 the bending strength of sample 6MX1 is higher by more than 15% at 28 days.

### 3.2. Compressive Strength

**Figure 3:**
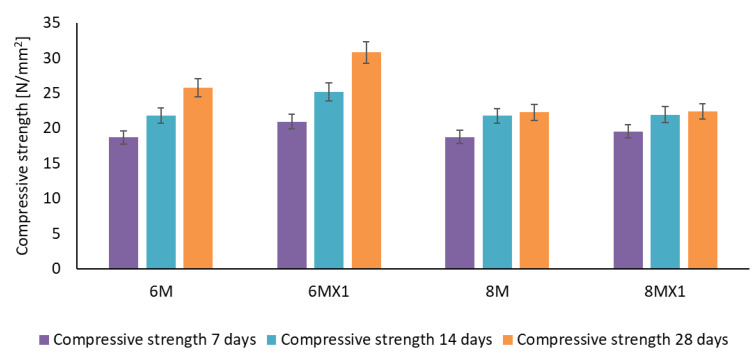
Compressive strength of the alkali-activated geopolymer samples.

The evolution of the compressive strength of the samples obtained at different test intervals is shown in Figure 3.

It can be seen that the compressive strength for all samples increases with the age of testing. It is also observed that for a molarity of 6M of NaOH solution, the sample (6MX1) containing 1% Fe_2_O_3_ and 1% MgO added to the ash used has a compressive strength more than 11% and 15%, respectively, 19% higher than the sample (6M) not containing these additional elements, at the age of 7, 14 and 28 days.

The evolution of the compressive strength of the samples obtained using a NaOH solution of molarity 8 is almost identical whether 1% Fe_2_O_3_ and 1% MgO was added or not (increase of less than 1% for ages 14 days and 28 days after casting). The most significant increase in compressive strength due to the addition of iron and magnesium oxides in the case of 8M NaOH solution molarity is recorded early, at the age of 7 days after casting (4%).

The compressive strength of sample 6MX1 at the test age of 28 days has the highest value being more than 37% higher than samples 8M and 8MX1. The increase in compressive strength can also be attributed to the microcrack filling effect of Fe_2_O_3_ and MgO acting as inactive granular fillers by the presence of unreacted phases (hematite, forsterite and periclase) in the geopolymer specimens [50].

The increase in mechanical strength, with the increase in molarity of the NaOH solution used in the preparation of the alkaline activator, could be attributed, in addition to the maturation in time and the completion of the geopolymer reactions, to the contribution of Na+ ions and OH- groups provided by the alkaline activator. Thus, in accordance with the literature, with references on the main steps of the geopolymerization mechanism, it is appreciated that both Na+ ions and hydroxyl groups play an important role in the dissolution and hydrolysis processes, breaking the bonds existing in the Si and Al source raw materials, contributing to produce Si-O-Al bonds, bonds known as geopolymer precursors [51]. More specifically, Na+ ions contribute to the balancing of negative charges produced by Si-O-Al formation, while OH ions play an essential role in the hydrolysis process of the geopolymer, producing the geopolymerization reaction and the formation of the aluminosilicate network with stable, stronger bonds, which ultimately lead to better mechanical strength of the material. This assessment is also in agreement with Khale and Chaudhary who appreciate that to obtain good mechanical strengths of the geopolymer binder it is necessary to identify an optimal molarity of NaOH, which, by providing Na+ ions, contributes to balance and optimize the geopolymerization reactions, reactions strongly dependent on the oxidative nature of the raw materials [52]. On the other hand, an excess of Na+ ions or hydroxyl groups, resulting from a too high molarity of the NaOH solution used in the preparation of the alkaline activator, will result in an early precipitation of the aluminosilicate gel, respectively, a reduction of the geopolymer specific bonds and, consequently, a reduction of the mechanical strengths [53,54,55]. In the present case, the experimentally recorded values indicate that with the addition of iron and magnesium oxides, this positive influence of the NaOH solution size on the mechanical strength is not necessarily maintained. The best situation is identified as sample 6MX1, the influence of the addition of oxides being weaker than the influence of the molarity of the NaOH solution, the microstructural analysis presented below providing elements to support this hypothesis.

**Figure 4:**
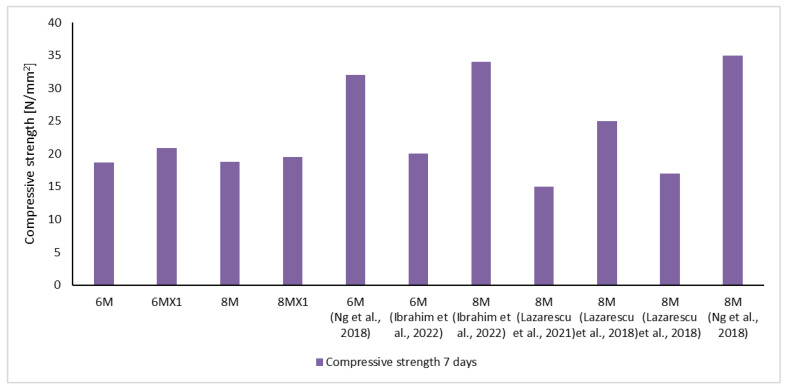
Comparative analysis of experimental results with literature reports.

Quantitatively analysing the results obtained in terms of the mechanical strengths, it can be said that they are in agreement with some specifications in the literature and even exceed other reported results, Figure 4. This observation can be explained on the one hand based on the contribution of source elements for the geopolymerization reaction, i.e., the oxide composition of fly ash, and on the other hand on the basis of the differences in the grain size of the raw material, an element which by its influence on the specific reactive surface that influences the kinetics of the geopolymerization reactions.

This is due to the fact that geopolymer materials are very sensitive to the physical and chemical characteristics of the raw material, which is the reason why there are situations when mixtures, which although prepared with the same NaOH solution molarity, but using fly ash with different chemical/oxide composition or grain size (specific characteristics of the raw material source) or situations where the solution is prepared from NaOH flakes or pearls, the physico-mechanical performances are very different (Figure 4).

**Figure 5:**
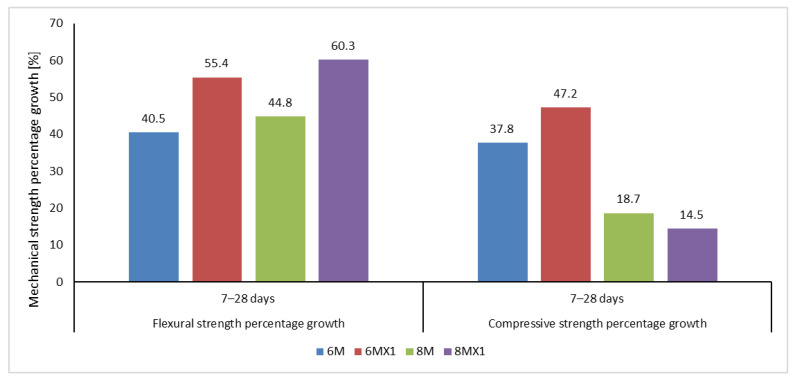
Percentage increase in flexural strength and compressive strength over 7–28 days after casting.

As can be seen from Figure 5, from the point of view of the evolution of the mechanical strengths, with the increase of the age of the specimens, there is also an increase of these parameters. It can also be seen that, in the case of the flexural tensile strength, this improvement is more evident in the 7–28 days period, while the compressive strength increases to a lesser extent in this period, a sign that, according to the literature, in the first days after preparation geopolymer binders tend to reach even 90% of the final compressive strength.

### 3.3. Scanning Electron Microscopy and Energy Dispersive Spectroscopy Analysis

**Figure 6:**
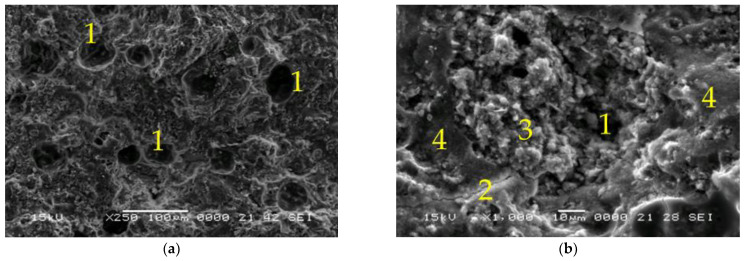
SEM micrographs of: (**a**,**b**) Sample 6M, ×250, respectively, ×1000 magnification (1—pores, 2—microcracks, 3—partially reacted fly ash and 4—dense zone of reacted fly ash).

**Figure 7:**
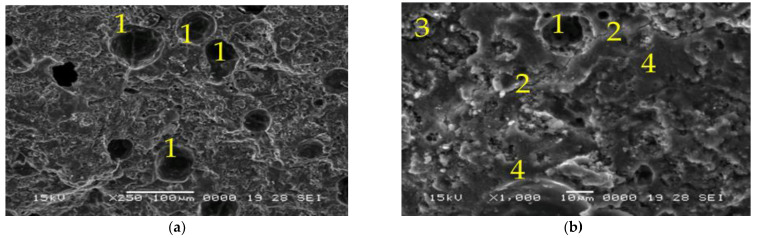
SEM micrographs of: (**a**,**b**) Sample 6MX1, ×250, respectively, ×1000 magnification (1—pores, 2—microcracks, 3—partially reacted fly ash and 4—dense zone of reacted fly ash).

**Figure 8:**
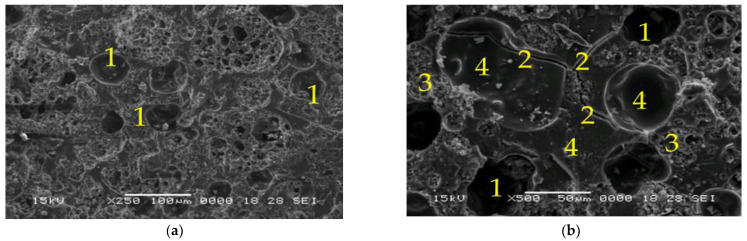
SEM micrographs of: (**a**,**b**) Sample 8M, ×250, respectively, ×1000 magnification (1—pores, 2—microcracks, 3—partially reacted fly ash and 4—dense zone of reacted fly ash).

**Figure 9:**
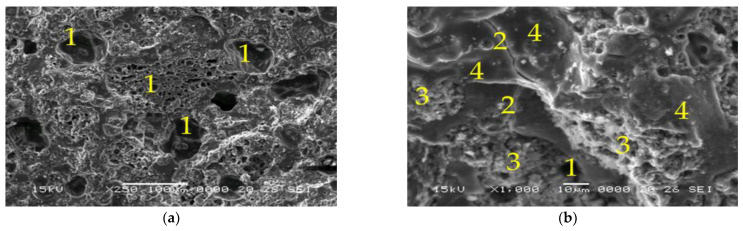
SEM micrographs of: (**a**,**b**) Sample 8MX1, ×250, respectively, ×1000 magnification (1—pores, 2—microcracks, 3—partially reacted fly ash and 4—dense zone of reacted fly ash).

Figure 6, Figure 7, Figure 8 and Figure 9 represent the SEM micrographs for the studied samples after 28 days of curing. These show the presence of pores (1), partially reacted raw materials (3), microcracks (2) and compact areas where the raw materials have fully reacted (4). The difference between the microstructure of samples 6M and 6MX1 is the portion of the geopolymer matrix and the amount of unreacted fly ash (Figure 6 and Figure 7). It can be seen that sample 6MX1 has a more compact geopolymer structure than 6M. The larger pore size of sample 6M and the larger amount of partially reacted fly ash were part of the reasons why its mechanical strength was lower than that of sample 6MX1.

The SEM micrographs for samples 8M and 8MX1 shown in Figure 8 and Figure 9 reveal that the pore number and microcracks size are higher compared to samples 6M and MX1. The higher number and size of pores may also be due to the higher molarity of the NaOH solution leading to an energetic reaction between the alkaline activator and the ash. Microcracks may be due to shrinkage during curing [56,57], exothermic dissolution of activators [58] and heat treatment. Although the molarity of 8M and 8MX1 is higher than that of 6M and 6MX1 samples, the presence of high pore numbers and high amount of unreacted ash has the effect of lowering the mechanical strengths of these samples.

**Figure 10:**
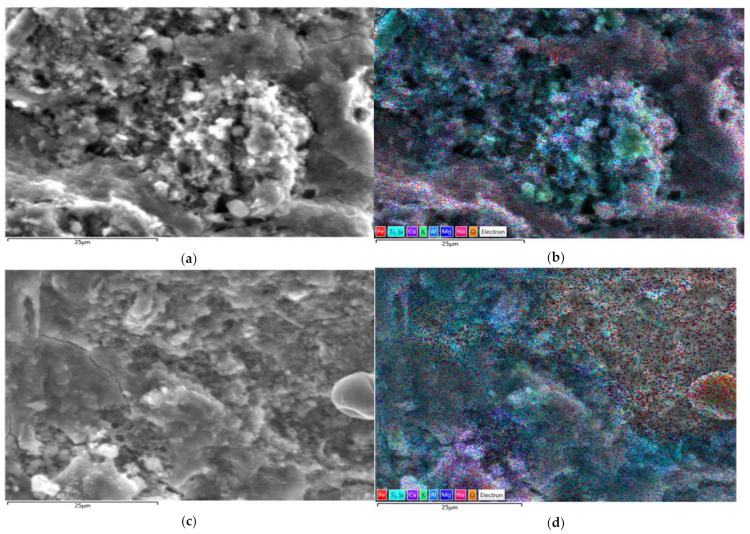
EDS analysis for sample 6M: (**a**) SEM image of selected area and (**b**) EDS stratified image of selected area; for sample 6MX1: (**c**) SEM image of selected area and (**d**) EDS stratified image of selected area.

**Figure 11:**
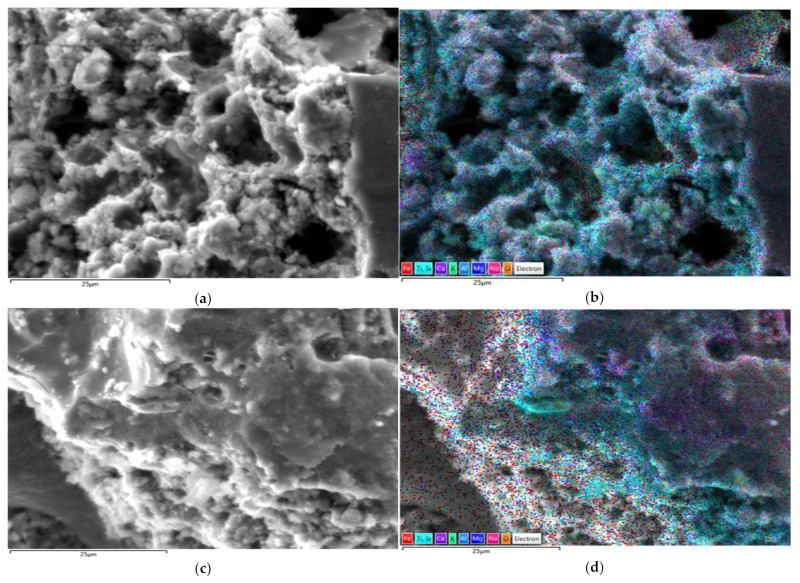
EDS analysis for sample 8M: (**a**) SEM image of selected area and (**b**) EDS layered image of selected area; for sample 8MX1: (**c**) SEM image of selected area and (**d**) EDS layered image of selected area.

Figure 10 and Figure 11 show the EDS images with the distribution of elements in the selected areas for the studied samples, and Table 3 gives the percentages of elements present in the samples.

The literature states that in EDS analysis for Si/Al ratio between 2.39 and 2.84 and for Na/Al between 0.34 and 0.53, respectively, the major reaction product is formed by alkaline hydroaluminosilicates of the zeolitic type in which Ca incorporation is also assumed. Additionally, for sufficiently high Si/Al ratio of 2.49 and a fairly high Na/Al ratio of 2.27, a carbonate variety of sodium and calcium hydroalumino-silicates is obtained [59]. Based on these clarifications and in accordance with the experimental results obtained and presented in Table 3, it can be stated that in the case of the studied samples (6M, 6MX1, 8M and 8MX1) the majority reaction product consists of zeolitic type hydroalumino-silicates and with slight tendencies to form carbonate variety of sodium and calcium hydroalumino-silicates.

Analysing Figure 10d and Figure 11d, areas where Fe_2_O_3_ is unreacted are noticed, which functions as a crack filler, while this could be a possible explanation for both the improvement in compressive strength and the macroscopic reddish colour identified in the tested specimens.

Analysing the ratio of the identified concentration of Fe and Al, respectively, Na, according to the values presented in Table 3, it is observed that the Fe/Al ratio varies in the range 0.4–0.7, and the Fe/Na ratio varies in the range 0.5–0.8, always the ratio characteristic of the mixture recipe with the addition of Fe_2_O_3_ and MgO being higher, both for both molarity of the NaOH solution used in the preparation of the alkaline activator. This trend is also considered to be a possible explanation for the higher mechanical strengths in the samples recorded for samples prepared with the addition of oxides.

### 3.4. X-Ray Diffraction (XRD) Analysis

**Figure 12:**
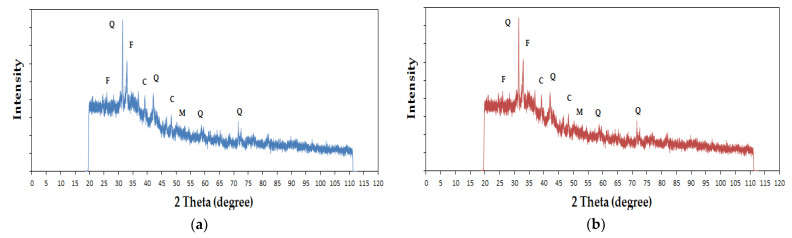
XRD analysis of geopolymer binders for (**a**) sample 6M and (**b**) sample 6MX1, where Q represents quartz, F—feldspar, C—calcite and M—mullite.

**Figure 13:**
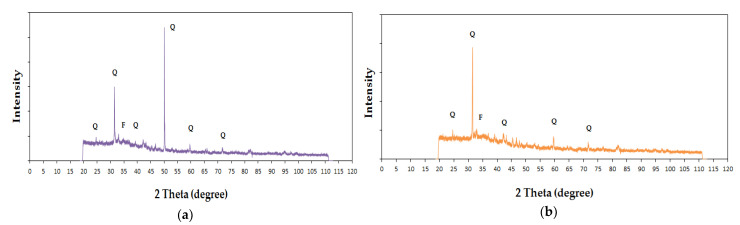
XRD analysis of geopolymer binders for (**a**) sample 8M and (**b**) sample 8MX1, where Q represents quartz, F—feldspar.

Analysing the X-ray spectra for samples 6M and 6MX1 in Figure 12, the presence of quartz, feldspar, calcite and mullite is observed, and for samples 8M and 8MX1 in Figure 13, the presence of quartz and feldspar in the geopolymer is observed.

Analysing Figure 12a and Figure 13a, it can be seen that the peaks with maximum intensities were for quartz, with the peak for the highest intensity 2θ maximum at 32° for sample 6M, respectively, at 50° for sample 8M, under the conditions of maintaining also an obvious peak at the 32° angle in the case of this sample characterized by a higher molarity of the NaOH solution used in the preparation of the alkaline activator. This displacement of the maximum 2θ angle is considered to be an indicator for preferential directions of crystallization, depending on the molarity of the NaOH solution used to prepare the alkaline activator. Comparing Figure 12b and Figure 13b, it is observed that the same major peak is identified for quartz at the maximum 2θ at 32° angle, but with a much higher intensity for the case of sample 8MX1, suggesting again the influence of the molarity of the NaOH solution used in the preparation of the alkaline activator on the crystallization mechanism. Comparing the samples preprepared with 6M NaOH solution, with and without the addition of Fe_2_O_3_ and MgO, no major differences in terms of the crystallization angles are identified (Figure 12). On the other hand, with increasing molarity of the NaOH solution to 8M, between the characteristic spectra of the samples prepared without, respectively, with addition of Fe_2_O_3_ and MgO, it is observed the maintenance of the characteristic quartz angle, 2θ, at 32°, but of a much higher intensity for the sample prepared with addition of oxides, concomitant with the maintenance of the characteristic feldspar peaks. In the literature it is stated that for a higher amount of iron trioxide and magnesium oxide added to fly ash the presence of hematite (Fe_2_O_3_), periclase (MgO) and forsterite (MgFeSiO_4_) mineral phases is observed in the X-ray spectrum [60], which in the present cases has not been confirmed.

From the study, it can be stated that iron trioxide, magnesium oxide and the molarity of the sodium hydroxide solution used in the preparation of fly ash-based geopolymer paste influence the physico-mechanical properties of the obtained heat-treated samples.

The bending tensile strength and compressive strength of the 6MX1 sample (containing iron trioxide, magnesium oxide and for which a molarity of 6M sodium hydroxide solution was used) had higher values compared to the other samples. This observation can be interpreted as a signal that for the specific case of fly ash with the oxide composition shown in Table 1, the most favourable case for obtaining the geopolymer binder would be the use of an alkaline activator prepared with 6M NaOH solution.

This increase in mechanical strengths can be explained by the action of iron trioxide which causes the formation of ferro-sialate groups and by the action of magnesium oxide which reduces the shrinkage of the sample. Additionally, the lower molarity of the hydroxide solution results in a less energetic reaction and fewer pores in the sample.

SEM micrographs reveal areas with fewer pores and fewer cracks for samples obtained with lower molarity of sodium hydroxide solution and smaller pore size, provided, however, that sufficient Na+ and OH− ions are available to allow good dissolution and extraction of Al and Si atoms from the raw material.

XRD analysis shows the presence of quartz, calcite, feldspar and mullite in samples obtained with a molarity of 6 of sodium hydroxide solution, and quartz and feldspar in samples obtained with a molarity of 8M. The formation of these elements is also influenced by the type of ash used and its chemical composition, and the addition of Fe_2_O_3_ and MgO leads to a preferential crystallization directive especially for quartz.

Identifying quartz (hardness 7, trigonal crystallization system), mullite (hardness 6/7.5, orthorhombic crystallization system), feldspar (hardness 6/6.5, tri- or monoclinic crystallization system) and calcite (hardness 3, trigonal or triclinic crystallization system), the following is estimated:

- The hardness of the crystallites as well as the specific crystallization system directly influences the compressive strength of the material;
- In the crystallite contact zone, for the crystallite combinations identified in the geopolymer material prepared with 6M NaOH solution, the cohesive energy would be higher than the cohesive energy specific to the intercrystallite contact zone of the geopolymer prepared with 8M NaOH solution.

The layered EDS images and the provided data reveal that in the case of the studied samples (6M, 6MX1, 8M and 8MX1) the majority reaction product is formed by zeolitic-type hydroaluminosilicates and with slight tendencies to form carbonate varieties of sodium and calcium hydroaluminosilicates. Iron trioxide and magnesium oxide are also observed to have a microcrack filling effect, i.e., they act as inactive granular fillers.

The mechanical strengths of the samples obtained, comparable to Portland cement, justify the use of these geopolymer pastes in the production of geopolymer concretes and in the production of precast concrete. Results obtained in the current study are in accordance with results previously obtained in the literature, while completing the knowledge about the production of alkaline-activated geopolymer materials [61,62,63,64,65,66,67,68,69,70,71,72,73,74,75].

## 4. Conclusions

The aim of this experimental study was to investigate the influence of the addition of Fe_2_O_3_ and MgO, respectively, and the influence of the molarity of the NaOH solution used in the preparation of the alkaline activator, on the mechanical strengths of the geopolymer binder prepared using locally sourced fly ash. Based on the obtained results, the following conclusions can be drawn:

1. The compressive and flexural strength of the 6MX1 specimen is higher than the other specimens (6M, 8M and 8MX1).
2. SEM micrographs reveal areas with fewer pores and fewer cracks for samples obtained with lower molarity of sodium hydroxide solution and smaller pore size.
3. XRD analysis shows the presence of quartz, calcite, feldspar and mullite in samples obtained with a molarity of 6M of sodium hydroxide solution, and quartz and feldspar in samples obtained with a molarity of 8M.
4. The EDS data show that the major reaction product is formed of zeolitic-type hydroaluminosilicates with slight tendencies to form carbonate varieties of sodium and calcium hydroaluminosilicates.
5. The addition of Fe_2_O_3_ and MgO to a geopolymer improves its physico-mechanical properties.

This paper contributes to the research developed so far worldwide on alkali-activated geopolymer materials with the following:

- The chemical, oxidic and mineralogical composition of the raw material used (flz ash) is specific only to the main source from which was provided and, according to the literature, has a major influence on the physico-mechanical characteristics of the geopolymer matrix;
- The NaOH solution used to prepare the alkaline liquid was prepared with local raw materials;
- Although some specifications in the literature analyse the influence of Fe and Mg oxides on the performance of geopolymer materials, in this case, these oxides do not represent the input of the basic raw material (fly ash), but are introduced as a controlled addition;
- The mix-design ratio and production technology are obtained following the analysis of literature but customized to the availability of resources and equipment. It is known that reproducibility is strongly influenced by the particularities of the materials and production techniques.

All these specific elements represented both challenges and risks, but also elements of novelty in the development of the experimental programme.

In the future, it is important to determine the optimum molar concentration of the NaOH solution used for the preparation of the alkaline activator and the optimal temperature range for obtaining samples with higher mechanical strengths.

## Figures and Tables

**Table 1:** Fly ash chemical composition.

**Oxides**	SiO_2_	Al_2_O_3_	Fe_2_O_3_	CaO	MgO	SO_3_	Na_2_O	K_2_O	TiO_2_	L.O.I
**F.A %**	46.9	23.8	10.1	10.7	2.7	0.5	0.6	1.7	0.9	2.1

**Table 2:** Mass ratio of materials used in the production of the mixtures.

Sample (NaOH conc.)	$m_{FA}\;(g)$	$m_{Fe_{2}O_{3}}\;(g)$	$m_{MgO}\;(g)$	$m_{sol}\;(g)$	$\frac{m_{Na_{2}SiO_{3}}}{m_{NaOH}}$	$\frac{m_{sol}}{m_{dry}}$
6M (6M)	267			240.30	2	0.9
6MX1 (6M)	267	2.67	2.67	245.10	2	0.9
8M (8M)	267	-	-	240.30	2	0.9
8MX1 (8M)	267	2.67	2.67	245.10	2	0.9

**Table 3:** EDS data for selected zones corresponding to samples 6M, 6MX1, 8M and 8MX1.

6M	Element	O	Si	Al	Na	Fe	Ca	K	Mg	Ti
	Weight%	44.9	27.4	10.1	7.9	4.1	2.4	1.5	1.2	0.5
	σ	0.2	0.1	0.1	0.1	0.1	0.1	0.0	0.0	0.1
6MX1	Element	O	Si	Al	Na	Fe	Ca	K	Mg	Ti
	Weight%	40.1	27.1	9.6	8.1	5.7	4.6	2.3	1.8	0.7
	σ	0.2	0.2	0.1	0.1	0.2	0.1	0.1	0.1	0.1
8M	Element	O	Si	Al	Na	Fe	Ca	K	Mg	Ti
	Weight%	42.0	28.5	9.4	9.2	4.8	2.0	2.1	1.2	0.7
	σ	0.2	0.2	0.1	0.1	0.1	0.1	0.1	0.0	0.1
8MX1	Element	O	Si	Al	Na	Fe	Ca	K	Mg	Ti
	Weight%	41.7	25.2	8.9	8.1	6.5	3.8	2.7	2.8	0.4
	σ	0.2	0.2	0.1	0.1	0.2	0.1	0.1	0.1	0.1

## Data Availability

Not applicable.
